# Chromatin in 3D: progress and prospects for plants

**DOI:** 10.1186/s13059-015-0738-6

**Published:** 2015-08-21

**Authors:** Chang Liu, Detlef Weigel

**Affiliations:** Department of Molecular Biology, Max Planck Institute for Developmental Biology, Tübingen, Germany

## Abstract

Methods that use high-throughput sequencing have begun to reveal features of the three-dimensional structure of genomes at a resolution that goes far beyond that of traditional microscopy. Integration of these methods with other molecular tools has advanced our knowledge of both global and local chromatin packing in plants, and has revealed how patterns of chromatin packing correlate with the genomic and epigenomic landscapes. This update reports recent progress made in this area in plants, and suggests new research directions.

## Introduction

Some time ago, cytological studies not only showed that chromosomes are arranged in species-specific ways during interphase but also suggested that chromosome length is an important determinant of overall chromosome conformation in the nucleus [1]. Some plant species have chromosomes that are several hundred megabases long, and these are often found in the ‘Rabl’ configuration [2], with centromeres and telomeres at opposite poles of the nucleus [3]. By contrast, short chromosomes tend to be arranged in a rosette configuration, such that the chromosome arms loop out from chromocenters that contain the densely packed centromeres [4]. In the model plant *Arabidopsis thaliana*, which has a small genome with chromosome arms of around 10 megabases, the positioning of genes within the nucleus can affect their expression levels [5]. The physical clustering of alleles that has been observed in *A. thaliana* [6] also suggests that genes are not randomly arranged within the chromosomes.

In non-plant species, the application of a 3C (chromatin conformation capture)-based method named Hi-C has greatly advanced our understanding of genome packing. In particular, Hi-C has revealed that TADs (topologically associating domains) are a predominant structural feature in most organisms [7–12]. Each TAD is a relatively isolated local unit, such that chromatin contacts within one TAD are generally preferred over those between different TADs. The locations of TAD boundaries are strongly correlated with local gene expression, epigenetic landscape and, where this had been tested, the binding of various insulator proteins (reviewed in [13, 14]). Here, we summarize what was previously known about nuclear chromosome arrangement in plants, and discuss how the recent application of 3C and related methods has provided a more detailed picture of chromatin packing in plants.

## Importance of local three-dimensional structure: chromatin loops

Within the DNA sequence of a chromosome, transcriptional enhancers can often be located far away from transcription units, but chromatin loops can bring distal regulatory elements into direct contact with the promoters that they control. The first plant chromatin loop to be found using the 3C method was at the maize *b1* gene, which controls pigmentation. Several additional loops have recently been found at four *A. thaliana* genes that have roles in flowering and hormone signaling [15–20].

The *b1* gene in maize encodes a transcription factor that regulates anthocyanin pigment production. Two epialleles of the *b1* gene, *B-I* and *B*′, are distinguished by their level of RNA expression, which is much higher in *B-I* than in *B*′. In husk tissues specifically, both epialleles form chromatin loops between the gene body and an enhancer located 100 kb upstream [15]. In the *B-I* allele, this enhancer has open chromatin that is thought to induce the formation of a multiloop structure between the *b1* transcription start site (TSS) and additional upstream regions that mediates high *b1* expression. By contrast, the *B*′ enhancer has compact chromatin, which prevents the formation of a multiloop structure and ultimately results in low *b1* expression [15].

The flowering repressor *FLOWERING LOCUS C* (*FLC*) of *A. thaliana* has emerged as an ideal platform for the discovery of many epigenetic regulatory mechanisms, involving histone modifications, small RNAs and long non-coding RNAs [21]. In recent work, the 5′ end of the *FLC* transcribed region was found to interact with sequences immediately downstream of the polyadenylation signal [16]. In non-plant organisms, chromatin loops connecting the 5′ and 3′ ends of genes have been proposed to support transcription by facilitating the recycling of RNA polymerase at actively transcribed genes [22], or by promoting the recruitment of RNA polymerase to reactivate gene expression [23]. Although the loop at the *FLC* gene is comparable to examples outside plants, its formation does not directly correlate with RNA expression: it can be detected in a wide range of genetic backgrounds that have very different levels of *FLC* expression. Its disruption does appear, however, to be an early response to prolonged cold exposure, or vernalization, which ultimately causes stable, Polycomb-mediated repression of *FLC* [16]. Disruption of the *FLC* loop relies on SWI/SNF chromatin-remodeling activity, as inferred from knockdown of the SWI-SNF subunit BAF60, which stabilizes the *FLC* chromatin loop [24].

Regulation of *FLC* involves the long noncoding RNA (lncRNA) *COOLAIR* [25]. It has been suggested that disruption of the *FLC* chromatin loop allows access of RNA polymerase II or of transcription factors to *COOLAIR* regulatory elements, which (through an intricate series of further events) ultimately leads to Polycomb-mediated silencing of *FLC* [16]. This scenario is similar to that proposed for the *PINOID* (*PID*) auxin-response gene, which has a chromatin loop in its promoter region [20]. This loop appears to preclude the binding of activating transcription factors, and with the loop present, *PID* expression is low. Formation of the loop depends on LHP1, an H3K27me3-binding plant homolog of HETEROCHROMATIN PROTEIN 1 [26, 27]. The promoter-distal end of the loop is densely methylated, and it contains the TSS of a lncRNA, *APOLO* (*AUXIN-REGULATED PROMOTER LOOP*), which is divergently transcribed relative to *PID*. Auxin stimulation induces DNA demethylation at the *APOLO* locus, which reduces the frequency of H3K27me3 marks and consequently LHP1 binding, and thereby leading to the opening of the loop. Simultaneously, *APOLO* expression is activated, which ultimately triggers a new round of RNA-dependent DNA methylation (RdDM) and deposition of associated H3K27me3 chromatin marks that repress the expression of *PID*. The full model thus suggests an elegant mechanism for transient induction of *PID*: auxin induces DNA demethylation, leading to ejection of LHP1 and disruption of the chromatin loop and thus activation of *PID* expression. Because *APOLO* lncRNA expression is activated at the same time, a new round of RdDM is initiated, leading to *PID* downregulation.

The flowering gene *FLOWERING LOCUS T* (*FT*) integrates many different environmental cues, including vernalization (by virtue of being a direct *FLC* target), photoperiod, age and ambient temperature. Much of this integration occurs at the *FT* locus itself, and its regulatory sequences are accordingly complex, as is the pattern of *FT* expression during the life cycle of the plant (reviewed in [28]). Sequences both upstream and downstream of the transcription unit, as well as intergenic elements, contribute to *FT* transcriptional regulation. One of these elements, an enhancer located 5.3 kb upstream of the transcribed region, makes contact with the TSS [18, 19]. This enhancer contains a CCAAT motif, which is typically bound by Nuclear Factor Y (NF-Y) transcription factors (also known as HAP, AnCF or CBF proteins). Some NF-Y proteins have been shown to bind to CONSTANS (CO) [29], a B-box factor that interacts with the element near the TSS and activates *FT* expression [30]. These results illustrate a typical scenario in which chromatin looping is a consequence of the interaction of enhancer-regulatory factor complexes with promoter-proximal sequences [18]. The *FT* homolog *TERMINAL FLOWER 1* (*TFL1*) is regulated very differently than *FT*, but also has complex regulatory sequences that include an enhancer located downstream of the transcription unit [31]. Binding of this enhancer by a complex of MADS-domain transcription factors causes it to dissociate from the TSS, and in turn, the disappearance of this loop appears to cause reduced expression of *TFL1* [17].

## Global chromatin packing in plants

**Fig. 1:**
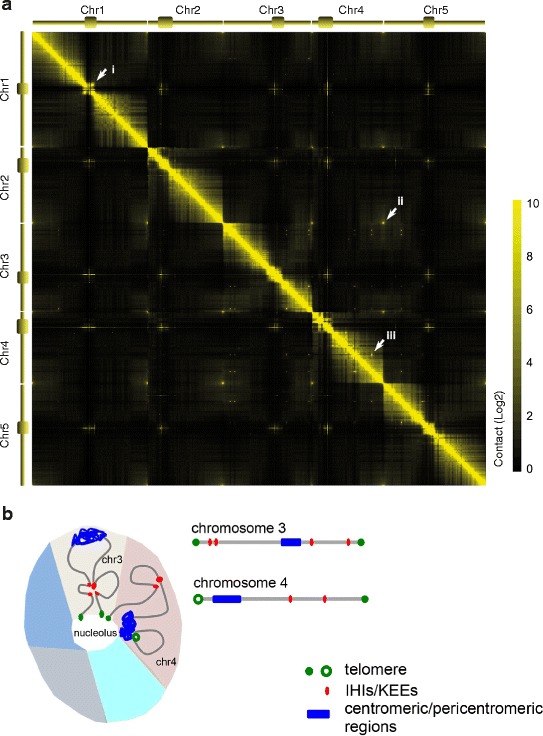
Chromosome packing in interphase nuclei of *Arabidopsis thaliana* seedlings. **a** A genome-wide interaction map of A. *thaliana* seedlings at 20-kb resolution. The normalized contact strength is shown with color gradient. For all chromosomes, the intra-chromosomal contact decreases as a function of genomic distance. *Arrows* highlight selected features. (*i*) The centromeric and pericentromeric regions are tightly packed and have few interactions with regions located on chromosome arms. This corresponds to general observations that centromeric regions appear as bright dots in *A. thaliana* nuclei stained with DAPI (4′6-diamidino-2-phenylindole dihydrochloride). On the other hand, in Hi-C maps of mutant plants where heterochromatin is decondensed, such as *met1*, *ddm1*, *suvh4*, *suvh5 suvh6*, and *atmorc6*, pericentromeric regions interact less with each other and more with the chromosome arms [32, 39]. (*ii*) Telomeres from different chromosomes are close to each other. According to fluorescent in situ hybridization (FISH) studies, telomeres often gather around the nucleolus [4]. (*iii*) Strong interaction among a subset of interstitial heterochromatin regions. These interactions have been captured by four independent Hi-C experiments [32–34, 39]; some of these interactions have also been seen with FISH [32, 33]. This Hi-C map is reproduced from our previously published interaction matrix [34]. **b** Diagram summarizing the conformation of interphase *A. thaliana* chromosomes with packing inferred from cytological and Hi-C experiments. Further details are shown for chromosomes 3 and 4. Every chromosome occupies a distinct territory (five differently colored territories are shown in this nucleus cross-section), but the relative positioning of the chromosomes within nuclei is largely random [59]. The tightly packed centromeric/pericentromeric portion (chromocenter) of every chromosome is often located close to the nuclear periphery or the nucleolus [4]. Chromocenters can fuse randomly, which produces strong inter-chromosomal interactions on the Hi-C map. Telomeres often cluster around the nucleolus, except for those close to nucleolus-organizing regions (NORs) on chromosomes 2 and 4 (not shown in this diagram), which frequently associate with their respective chromocenters [4]. The interactive heterochromatic island (IHI)/KNOT engaged element (KEE) regions form strong intra-chromosomal, and sometimes inter-chromosomal contacts (not shown in this diagram), and are readily spotted on Hi-C maps

Like studies of animals and humans, the plant field has begun to go beyond the analysis of chromatin loops at individual loci and is rapidly adopting the 4C and Hi-C genome-wide methods [32–35]. At the chromosomal level, Hi-C maps generated from *A. thaliana* seedlings have revealed patterns that correspond well with cytological observations (Fig. 1).

At the megabase scale, the *A. thaliana* chromosome arms are partitioned into structural domains that can be classified as either compact or loose [33]. The correlation of this domain structure with the epigenetic landscape is partly reminiscent of that of the ‘AB compartment’ originally discovered in human Hi-C data [36]. Formation of these large-scale domains is unaffected in the *crwn1* (*crowded nuclei 1*) and *crwn4* mutants, which both have much smaller nuclei than wild-type plants, suggesting independence between nuclear morphology and chromosome packing [33]. At a more local scale, *A. thaliana* differs from most other organisms in that obvious TADs are not a predominant feature of *A. thaliana* genome organization. The lack of animal-like TADs in *A. thaliana* correlates with the absence of homologs of canonical insulator proteins such as CTCF [32, 34]. Moreover, experiments with transgenes have revealed very little, if any, credible evidence for insulator-like DNA sequences in *A. thaliana* [37]. Nevertheless, analysis of a high-resolution *A. thaliana* Hi-C map led to the identification of over 1000 TAD-boundary-like and insulator-like regions [34]. These regions have properties similar to those of sequences at the borders of animal TADs: there are limited chromatin contacts that cross these regions, and they are enriched for open chromatin and highly expressed genes [34], indicating a strong connection between transcription and local chromatin topology [33, 36]. These TAD-boundary-like and insulator-like regions were only noted after the resolution of *A. thaliana* Hi-C maps was increased from the 20 kb to the 2 kb range [34]. This is reminiscent of studies on *Saccharomyces cerevisiae*, in which TADs were only noticed when utilizing a high-resolution variant of Hi-C that uses DNA digestion by micrococcal nuclease instead of restriction enzymes [38].

Apart from more local interactions, there are prominent intra- and inter-chromosomal interactions among heterochromatic regions dispersed throughout the otherwise euchromatic chromosome arms [32–34, 39] (Fig. 1). These regions, named interactive heterochromatic islands (IHIs) [32] or KNOT engaged elements (KEEs) [33], range in size from 20–150 kb and are enriched for heterochromatic histone marks and transposons, even though they are not generally silenced. The mechanism by which these contacts are made remains unclear as similar features are found in other regions that do not behave as IHIs/KEEs. Interactions between IHIs/KEEs are largely unchanged even when most DNA methylation or heterochromatin H3K9me3 marks are removed [32]. These findings further suggest that DNA methylation and H3K9me2 do not directly cause the tethering of IHIs/KEEs.

The relationships between various chromatin modifications and chromatin packing have also been explored. H3K27me3, which is associated with Polycomb Repressive Complexes (PRCs), correlates with compact chromatin, and mutants lacking this histone mark have dramatically reduced chromatin contacts within such regions [32, 33]. H3K27me3 was also found to be enriched in ‘positive strips’, a special Hi-C feature apparent in a high-resolution *A. thaliana* Hi-C map [34]. Chromatin regions annotated as positive strips showed more frequent looping interaction with neighboring chromatin, thus forming contrasting lines of high contacts on the Hi-C map. These findings imply that, in addition to participating in local gene silencing, H3K27me3 might also directly or indirectly play a structural role in forming higher-order chromatin structure in plants.

## Challenges and outlook

Plant genomes are very diverse, and so are their three-dimensional (3D) structures [40]. *A. thaliana* has short chromosomes that adopt a rosette conformation. By contrast, species with long chromosomes feature what is known as the ‘Rabl’ conformation, and such differences are expected to be visible in Hi-C maps. Similarly, chromosomes can be quite differently organized, even in species that have similar chromosome number or genome size. The *A. thaliana* relatives *Arabidopsis lyrata* and *Capsella rubella* both have genomes that are about 50 % larger than that of *A. thaliana* [41, 42]. However, while genome expansion occurred mostly on the chromosome arms in *A. lyrata*, the increase in genome size in *C. rubella* is confined to the centromeres. It will be interesting to see how these differences are reflected in Hi-C maps of these species. Such closely related species that have rampant structural variation also afford a great opportunity to determine at a more fine-grained scale how deletions or insertions affect local chromatin–chromatin interactions.

The most impressive recent Hi-C study was the one by Rao and colleagues [43], who provided an extremely high-resolution map of chromatin contacts in human cells, based on an enormous amount of DNA sequence. Similarly high-resolution Hi-C maps are needed for *A. thaliana*, which has a very high gene density of about one gene per 5 kb. If local chromatin loops are as widespread in *A. thaliana* as they are in humans, many chromatin loops that have roles in the regulation of transcription would have a comparatively small size. The identification of such small loops is a technically and computationally challenging task. First, conventional 3C-based methods need to be coupled with additional steps to increase the sequencing depth of query regions, as this is a prerequisite to achieving a more accurate estimate of background signals or random chromatin interactions that are associated with loci of interest. Approaches that can help to provide this resolution include selective amplification-based methods, such as 4C and 5C [44–46], the hybridization-based CHi-C method [47], and the immunoprecipitation-based ChIA-PET method [48]. Micro-C, which uses micrococcal nuclease to digest DNA into nucleosomes, further improves the resolution of contact maps [38]. On the computational side, re-evaluating the systematic biases of Hi-C experiments, as noted by Yaffe and Tanay [49], might be necessary for the robust detection of small chromatin loops. For example, besides being a factor that influences the amplification efficiency of library molecules, GC content has been shown to correlate with short-range chromatin contact in mammals, probably as a direct consequence of the action of certain GC-rich elements [50]. Other biases that confound the identification of chromatin loops over short genomic distances, such as the distribution of restriction enzyme cutting sites, must also be considered [34].

To complement sequencing-based methods, there are cytological tools that can visualize and monitor the behavior of chromatin loci in the nucleus. For example, padlock fluorescent in situ hybridization (FISH) [51] in combination with photoactivated localization microscopy (PALM) [52] might be able to increase the resolution of traditional FISH, so that small chromatin loops can be detected directly. There are already several live imaging systems that can be used to observe chromatin in plants. For example, visually trackable T-DNA insertions have revealed an influence of mobility and subnuclear localization on local gene expression [5]. In another study, physical clustering of trackable *FLC-LacO* transgene loci was observed in connection with Polycomb-mediated silencing [6]. Both studies employed *LacO* arrays that can be recognized specifically by bacterial LacI protein labeled with fluorescent proteins. Today, more sophisticated genome-editing techniques such as CRISPR/Cas9 would allow the non-random insertion of *LacO* arrays into the genome. A CRISPR/Cas-based chromatin-imaging method has already been used in mammalian cell lines for visualization of non-repetitive genomic loci [53]. The recent development of a multicolor CRISPR labeling system further allows simultaneous tracking of different loci [54].

Many environmental and developmental factors, such as light intensity, temperature, microbe infection, and cell differentiation, can trigger global rearrangement of chromatin in plants [55–58], and we are looking forward to studies that will complete the rather coarse picture we have today by analyzing local chromatin topology at high resolution under different conditions and in specific cell types. In addition, we are excited about the possibility of placing such observations in an evolutionary context, as plant genomes are particularly dynamic, undergoing frequent genome expansions and contractions over very short time scales. Surely, such dramatic changes in genome size must be reflected in the 3D organization of the genome itself. An important question will be whether chromatin loops and other types of interactions can compensate for drastic changes in the linear size of the genome, so that regulatory elements can exert their effects independently of whether they are 2 or 20 kb from a promoter.

## Abbreviations

3C: Chromatin conformation capture
3D: Three-dimensional
*APOLO*: *AUXIN-REGULATED PROMOTER LOOP*
*crwn1*: *crowded nuclei 1*
FISH: Fluorescent in situ hybridization
*FLC*: *FLOWERING LOCUS C*
*FT*: *FLOWERING LOCUS T*
IHI: Interactive heterochromatic island
KEE: KNOT engaged elements
lncRNA: long noncoding RNA
NFY: Nuclear Factor Y
*PID*: *PINOID*
RdDM: RNA-dependent DNA methylation
TAD: Topologically associating domain
*TFL1*: *TERMINAL FLOWER 1*
TSS: Transcription start site
